# Unknown makes unloved—A case study on improving integrated health and social care in the Netherlands using a participatory approach

**DOI:** 10.1111/hsc.12901

**Published:** 2019-11-27

**Authors:** Manon Lette, Marijke Boorsma, Lidwien Lemmens, Annerieke Stoop, Giel Nijpels, Caroline Baan, Simone de Bruin

**Affiliations:** ^1^ Department of General Practice and Elderly Care Medicine Amsterdam Public Health Research Institute Amsterdam UMC – VU University Amsterdam Amsterdam The Netherlands; ^2^ Centre for Nutrition, Prevention and Health Services Research National Institute for Public Health and the Environment Bilthoven The Netherlands; ^3^ Scientific Centre for Transformation in Care and Welfare (Tranzo) University of Tilburg Tilburg The Netherlands

**Keywords:** case study research, health and social care, integrated care, interprofessional education and service developments, multi‐professional collaborations, participative research

## Abstract

Many initiatives integrating health and social care have been implemented in order to provide adequate care and support to older people living at home. Further development of existing initiatives requires iterative processes of developing, implementing and evaluating improvements to current practice. This case study provides insight into the process of improving an existing integrated care initiative in the Netherlands. Using a participatory approach, researchers and local stakeholders collaborated to develop and implement activities to further improve collaboration between health and social care professionals. Improvement activities included interprofessional meetings focussing on reflection and mutual learning and workplace visits. Researchers evaluated the improvement process, using data triangulation of multiple qualitative and quantitative data sources. According to participating professionals, the improvement activities improved their communication and collaboration by establishing mutual understanding and trust. Enabling factors included the safe and informal setting in which the meetings took place and the personal relationships they developed during the project. Different organisational cultures and interests and a lack of ownership and accountability among managers hindered the improvement process, whereas issues such as staff shortages, time constraints and privacy regulations made it difficult to implement improvements on a larger scale. Still, the participatory approach encouraged the development of partnerships and shared goals on the level of both managers and professionals. This case study highlights that improving communication between professionals is an important first step in improving integrated care. In addition, it shows that a participatory approach, in which improvements are co‐created and tailored to local priorities and needs, can help in the development of shared goals and trust between stakeholders with different perspectives. However, stakeholders' willingness and ability to participate in such an improvement process is challenged by many factors.

**What is known?**

- Integrated care is perceived as a promising solution to support older people living at home with complex needs.
- Although many integrated care initiatives exist, collaboration between health and social care professionals remains challenging.
- Integrated care is a complex process that is influenced by factors on multiple levels of the health and social care systems.

**What this paper adds?**

- Good relationships, trust and communication are important prerequisites to interprofessional collaboration.
- Participatory methods to improve integrated care allow for the development of partnerships and shared goals on multiple levels of the health and social care systems.
- Successful improvement processes require sufficient time, commitment of stakeholders on multiple organisational levels and a process facilitator.

## 1 INTRODUCTION

Due to demographic changes, health systems face the challenge of providing care and support to an increasing number of older people living in their home environments. As people age, they often encounter multiple health and social care needs. Meeting these needs requires health and social care professionals to collaborate in a proactive and coordinated manner, often called integrated care (de Bruin et al., 2018; World Health Organization, 2008, 2015). Over the last decades, a wide range of integrated care initiatives have been implemented, including, for example, initiatives on multidisciplinary community‐based care (Huss, Stuck, Rubenstein, Egger, & Clough‐Gorr, 2008; Van der Elst et al., 2018) or case management for older people (You, Dunt, Doyle, & Hsueh, 2012). Although evaluations have shown positive effects on outcomes such as healthcare utilisation, well‐being and patient satisfaction, evidence remains inconclusive (de Bruin et al., 2012; Hoogendijk, 2016; Hopman et al., 2016; Huss et al., 2008; Looman, Huijsman, & Fabbricotti, 2018; Martinez‐Gonzalez, Berchtold, Ullman, Busato, & Egger, 2014; Mayo‐Wilson et al., 2014; Ouwens, Wollersheim, Hermens, Hulscher, & Grol, 2005; Stall, Nowaczynski, & Sinha, 2014; Stokes et al., 2015; Stuck, Egger, Hammer, Minder, & Beck, 2002; Van der Elst et al., 2018; You et al., 2012). These inconsistent findings could be due to the heterogeneous nature of integrated care initiatives (Amelung et al., 2017; Busetto, Luijkx, & Vrijhoef, 2017), differences in outcome measures (Hoogendijk, 2016) or because implementation of these initiatives is a complex process (Mayo‐Wilson et al., 2014). Indeed, many enabling and constraining contextual factors regarding the implementation of integrated care initiatives have been identified, such as professionals' skills and motivation, organisational culture, funding or IT‐systems (Busetto, Luijkx, Calciolari, Ortiz, & Vrijhoef, 2018; Cameron, Lart, Bostock, & Coomber, 2014; Mackie & Darvill, 2016).

Because integrated care is complex and the implementation and effectiveness of integrated care initiatives is dependent on the local context within which it is implemented, it has been argued to view integrated care not as an intervention in itself that needs to be proven effective, but rather as a complex overarching strategy to change and innovate service delivery (Amelung et al., 2017). Change strategies, being iterative processes of implementation, evaluation and further refinement, require stakeholder involvement and continuous feedback on process and outcomes in order to learn from past experiences and thus to advance development (van Dongen et al., 2018; Greenhalgh, Robert, Macfarlane, Bate, & Kyriakidou, 2004). Therefore, evaluations of integrated care should not only focus on interventions' outcomes, but also on the process through which such changes happen (Eyre, Farrelly, & Marshall, 2017; Greenhalgh et al., 2004; Manojlovich, Squires, Davies, & Graham, 2015). Participatory research designs, which are characterised by effective partnerships between researchers and local stakeholders and facilitate the development and implementation of locally relevant knowledge, can therefore add a valuable perspective to research on integrated care (Eyre et al., 2017; Glasgow, 2013).

This case study aimed to use a participatory approach as a way to facilitate further improvement of integrated care. The study was part of a larger European research project called SUSTAIN, which investigated integrated care for older people and specifically focused on improving existing integrated care initiatives, rather than developing new ones (de Bruin et al., 2018). Another objective of the project was that improvements to these initiatives should be tailored to the needs of local stakeholders. This particular case study aimed to develop and implement such locally relevant improvements to an existing initiative in the north of the Netherlands. This paper describes this improvement process and the factors influencing it. Insight into these processes and factors provides lessons, which are transferable to other integrated care initiatives.

## 2 METHODS

### 2.1 Study design and setting

Following the SUSTAIN project's methodology and timeline (de Bruin et al., 2018), we used a case study design (Yin, 2009) to evaluate the improvement process of an existing integrated care initiative (‘case’) over the course of two and a half years. This case study focused on the West‐Friesland region in the Netherlands. Over the past decade, several services have been implemented within the region with the aim of improving care and support provided to older people living at home with complex needs. These services include (a) a proactive care model implemented among general practitioners (GPs) (Muntinga et al., 2012), (b) comprehensive case‐management for people with dementia and their caregivers (Glimmerveen & Nies, 2015) and (c) so‐called ‘social community teams’, in which municipalities collaborate with home care and social care organisations to deliver instrumental aid and social support. As such, many different organisations are involved in delivering care and support for older people living at home in West‐Friesland, which challenges integrated care.

**Figure 1:**
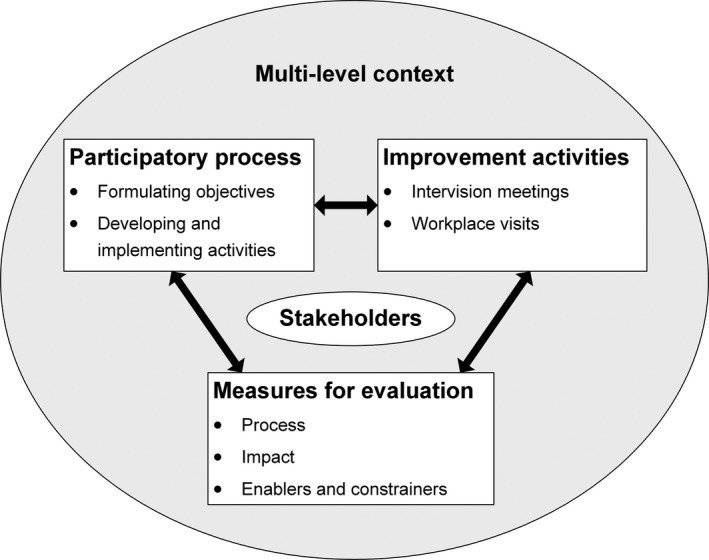
The improvement process guided by the Evidence Integration Triangle. This figure was adapted from Glasgow et al. (2012)

From November 2015 to April 2018, we used a participatory approach to facilitate the development and implementation of improvements to the way of working in West‐Friesland, in order to achieve better integrated care. This improvement process was guided by the Evidence Integration Triangle (EIT) (Glasgow, Green, Taylor, & Stange, 2012), and consisted of three interacting core elements: the *participatory process*, *improvement activities* and *practical measures* for evaluation (see Figure 1). The *participatory process* was characterised by collaboration between researchers and stakeholders to ensure the improvement process was tailored to local priorities and context. This participatory process resulted in *improvement activities* that were implemented to improve current practice, and *practical measures* were used to evaluate the improvement process. This evaluation focused both on impact of the improvement activities and on the participatory process itself, to gain an understanding of how and why these activities brought about change. The next sections will further elaborate on the practical application of these three core elements.

### 2.2 Participatory process and stakeholders

Several steps were taken to ensure the desired participatory approach was maintained during development and implementation of the improvement activities. First, members of the research team formed coalitions of stakeholders on multiple levels of health and social care in West‐Friesland. Table 1 shows the stakeholders on managerial level, from now on ‘steering group’, and operational level, from now on ‘professionals’, that participated in the improvement process. Professionals were employed by the same organisations that participated in the steering group. Additionally, one of the research team members (MB) was a former coordinator of elderly care in this region. Already known to and trusted by the steering group and professionals, she did not represent any specific organisation and could thus act as a neutral convenor between individual professionals, and between management and operational levels.

**Table 1:** Stakeholders participating in the improvement process

Steering group (managerial level)	Professionals (operational level)
Manager of home care organisation 1 Manager of home care organisation 2	Home care nurse 1 Home care nurse 2 Home care nurse 3
Manager of organisation providing integrated community care to people with dementia	Case manager for people with dementia 1 Case manager for people with dementia 2
Manager of social care organisation	Social worker
Policy officer from municipality	Municipality support consultant
General Practitioner	Geriatric practice nurse 1 Geriatric practice nurse 2 Geriatric practice nurse 3
Representative from regional advocacy organisation for older people	

The research team organised and facilitated regular steering group meetings, during which the research team and the steering group collaborated to identify potential areas for improvement, define shared objectives and develop improvement activities. Improvement activities were verified during an additional meeting with professionals in order to ensure improvements were tailored to their needs as well. The research team was responsible for data collection (see below under ‘*Measures and data collection*’) to evaluate process and impact and provide the steering group with feedback on this.

### 2.3 Improvement activities

Steering group members identified two main areas for improvement and formulated the following objectives:

1. It appeared to be difficult for professionals to truly put the needs and wishes of older people at the centre of their activities. The steering group aspired to enhance professionals' awareness regarding their own professional habits, and how these affect the person‐centredness of their way of working.
2. Available services were still very fragmented across different domains of health and social care. The steering group aspired to align communication and collaboration between professionals and increase their understanding of one another's roles and responsibilities.

Two improvement activities were developed and implemented among the professionals. The first comprised of regular ‘intervision meetings’. These are meetings in which peer supervision and methodical discussions help participants to reflect on their personal and professional development (Bellersen & Kohlmann, 2016). These meetings aimed to stimulate reflection on their professional habits in relation to the older people they cared for. Meetings were held once every two months during evening hours in an informal setting and included dinner. The meetings were organised by the principal researcher (ML) during which MB, who had experience with intervision methods, facilitated the process. The second improvement activity consisted of workplace visits, whereby professionals visited and shadowed each other during relevant parts of the day, in order to increase their understanding of one another's roles, responsibilities and expertise. The research team encouraged these visits during the intervision meetings, but the responsibility for organising these visits lay with the professionals.

### 2.4 Measures and data collection

Using a mixed‐methods approach, this case study used a combination of qualitative and quantitative data sources to collect data among steering group members and professionals participating in the improvement activities (see Table 2). Quantitative data sources included timesheets, which professionals used to record time spent on the improvement activities, and the Team Climate Inventory (TCI) (Kivimaki & Elovainio, 1999). The TCI assesses team climate and functioning on a scale from one to five, and includes subscales addressing team vision, participative safety, task orientation and support for innovation. Qualitative data sources included interviews with steering group members and a group interview with professionals. Interviews addressed participants' experiences with the implementation and perceived impact of the improvement activities, as well as enabling and constraining factors. In addition, we collected process data through minutes of meetings and researchers' field notes.

**Table 2:** Data sources, data collection moments and quantity of data collected per source

Data source	Objective	Collection	Quantity
Team Climate Inventory (TCI) (Kivimaki & Elovainio, 1999)	Measures changes in team coherence among steering group members and professionals	At start and end of implementation	Baseline: *n* = 11 respondents Follow‐up: *n* = 10 respondents
Interviews with steering group members	Provides perceptions and experiences of steering group members with regard to process, outcomes and contextual factors	At end of implementation	*n* = 4 participants
Group interview with professionals	Provides perceptions and experiences of professionals with regard to process, outcomes and contextual factors	At end of implementation	1 interview with *n* = 4 participants
Timesheets	Provides information on amount of time spent on intervention by professionals	Halfway and at end of implementation	*n* = 7 respondents
Minutes of steering group meetings	Provides information on processes, discussions and decisions during steering group meetings	During development and implementation	*n* = 4 meetings
Minutes of intervision meetings	Provides information on processes, discussions and decisions during intervision meetings	During implementation	*n* = 6 meetings
Field notes	Researchers notes on the process and progress of the improvement process	During development and implementation	Notes from a 30‐month period

Recruitment of participants took place during meetings, via email or through telephone. Of the seven steering group members who were approached, five completed a questionnaire and four participated in an interview. Of the 10 professionals who were approached, eight completed a questionnaire (either at baseline, follow‐up or both). Furthermore, eight professionals completed a timesheet and four participated in the group interview. Data for the questionnaires and time sheets were collected by mail or during meetings. Interviews with steering group members were conducted over telephone, whereas professionals were interviewed during a group interview.

The principal researcher took minutes of all steering group and intervision meetings, recording attendance, topics discussed and decisions made. Minutes were shared with participants to ensure they properly reflected the meetings. Field notes were recorded by the principal researcher throughout the improvement process. These notes included records of telephone calls, emails or one‐on‐one meetings with stakeholders, decisions made by the research team outside the steering group or intervision meetings and information about other relevant events in the region.

### 2.5 Data analyses

This case study used a triangulation approach in order to validate findings using multiple sources and types of data (Giacomini & Cook, 2000). Data were analysed in three steps:

- Each data source was analysed individually. Qualitative data were analysed according to the framework analysis method (Gale, Heath, Cameron, Rashid, & Redwood, 2013) using predefined code‐structures. The code‐structure for the interview transcripts was derived from the interview's topic list, whereas the code structure for both meeting and field notes were based on predefined themes identified in literature. Two researchers (ML and LL) independently coded the data, crosschecked each other's codes and discussed differences to reach consensus. Software for qualitative data analysis (MAXQDA 2018.0.5) was used to aid in the analysis by sorting data according to codes and themes. For quantitative data, different analytical approaches were used. Hours spent on the intervention were summed up to calculate a total score. Outcomes of the TCI were analysed with SPSS 24.0.0.1 by calculating and comparing mean scores.
- Findings from each individual data source were reduced to a series of thematic statements (for qualitative data) and summaries (for quantitative data).
- These thematic statements and summaries were amalgamated and subjected to a process of pattern‐matching across the data, in order to find patterns, themes and evidence that explained what worked when improving integrated care and which enabling and constraining factors influenced this process.

### 2.6 Ethics

The Medical Research Ethics Committee of the VU University Medical Centre in Amsterdam concluded that the Medical Research Involving Human Subjects Act (WMO) does not apply to this study (reference number 2016.507). Study participation was voluntarily and data collection took part upon informed consent.

## 3 FINDINGS

Findings are presented in four sub‐sections, that describe (a) the implementation and impact of the improvement activities, (b) the experiences with the participatory process, and the factors that (c) enabled and (d) constrained the improvement process undertaken in this case study. Where appropriate, we will refer to some illustrative quotes supporting our findings. Table 3 provides these quotes.

**Table 3:** Quotes from (group)interviews to illustrate findings

Quote #	Participant	Extract
1	Geriatric practice nurse 1	‘It's that you feel comfortable to ask someone, because you know it's something that is probably part of their job. Otherwise, you wonder sometimes about whom to go to. Now you know a little bit of what everyone is doing. And you know each other, so it's not so bad if you ask the wrong question to the wrong person sometimes. Because then the other one will just tell you, no, you should go to him or her with that question. That is that feeling of safety and trust that you have’.
2	Municipality support consultant	‘Definitely the collaboration with the others involved in my working area. Or OUR working area, I should say. Just that you know where to find each other’.
3	Manager of home care organisation 2	‘What I would have preferred to get out of the project was for us to formulate together what we actually expect from the [proactive primary care model previously implemented in the region] and how we would approach that in the community together, because then we would have had something that we could all make agreements on […] and then we would collectively commit to a model that would help us to get those older people at home in the picture. I think that would be more valuable overall compared to what we did now […]’
4	Manager of home care organisation 1	‘…[M]ost of all you see the divide between the doctors and the nurses versus social care and the municipality. Those really are two different worlds, and they have to grow towards each other. That's what I think was the beauty of this project’.
5	Manager of dementia care organisation	‘How I see it, from what I know, is that at least at the level of the people who are in charge, so the managers and the administrators, that these people have come to find each other better and better. Of course, there were other things going on in the region that supported this […]. But meeting each other for [this project] did definitely supported that, especially in terms of vision’.
6	Manager of home care organisation 2	‘I think that […] we've been searching for a long time for what it was that we would work on with each other, specifically. As I've experienced it, there would be nuances or we would suddenly be doing something different, or someone else would join the steering group which meant we were repeating a lot. Or people didn't come to the meetings or I didn't come myself. All in all, for me it never became specific enough’.

### 3.1 Implementation of the improvement activities

Six intervision meetings were held during a 12‐month period. Although 10 professionals were invited to participate, five of them dropped out throughout the course of the meetings due to lack of time and high caseload. The remaining five participants, being a practice nurse, a case‐manager, a home care nurse, a social worker and a municipality support consultant, attended meetings regularly. Professionals indicated to have spent a total of 102.5 hr on the project, most of which were allocated to attending intervision meetings. Some time was also spent on workplace visits. Three out of the five regular attendants to the meetings performed at least one workplace visit.

Initially, the intervision meetings aimed to target the steering group's first improvement objective regarding the person‐centredness of professionals' way of working. Meeting notes showed that responsiveness among professionals to address this aspect of their way of working was low, as they gave precedence to the need to explore and reflect on their relations to each other. After two meetings, the facilitators chose to adapt the meeting content, making it more flexible to address questions arising from the professionals. This meant that the focus of the intervision meetings shifted towards collaboration and communication between professionals. As such, the steering group's first improvement objective was not addressed as initially intended.

Regarding the second improvement objective, interviews with professionals and steering group members revealed that they thought the project had resulted in improved alignment between health and social care professionals. Steering group members indicated they experienced better working relationships and more trust on management level. Professionals explained that the intervision meetings allowed for professional development. According to them, both these meetings and the workplace visits increased their awareness of one another's roles, responsibilities and expertise. Professionals also felt the intervision meetings resulted in more understanding and trust, making it easier for them to collaborate (see Table 3, quote 1 and 2). TCI scores seemed to confirm these patterns found in the qualitative data (see Table 4). Although the number of respondents is small, overall scores improved during the implementation period, from a mean score of 3.3 (*SD* 0.84) at baseline to 3.6 (*SD* 0.62) at follow‐up.

**Table 4:** Mean scores on the Team Climate Inventory

	Baseline (mean; *SD*)			Follow‐up (mean; *SD*)		
	Total (*n* = 10)	SG (*n* = 4)	Profs (*n* = 6)	Total (*n* = 10)	SG (*n* = 4)	Profs (*n* = 6)
Total TCI score	3.3 (0.84)	3.1 (0.56)	3.3 (1.03)	3.6 (0.62)	3.8 (0.22)	3.4 (0.76)
Vision	3.6 (0.95)	3.8 (0.80)	3.5 (1.10)	3.7 (0.68)	4.0 (0.35)	3.5 (0.78)
Participative safety	3.1 (0.98)	2.8 (0.94)	3.4 (1.01)	3.7 (0.94)	4.1 (0.29)	3.5 (1.11)
Task orientation	2.9 (1.12)	2.6 (0.74)	3.1 (1.34)	3.3 (0.39)	3.2 (0.19)	3.3 (0.47)
Support for innovation	3.3 (0.75)	3.3 (0.32)	3.3(0.98)	3.4 (0.68)	3.4 (0.19)	3.3 (0.84)

Note

This table presents data on team climate collected among steering group members and professionals at the start and end of implementation of the improvement project. A more detailed table providing scores per participant may be found in Appendix S1.

Abbreviations: Profs, professionals; SD, standard deviation; SG, steering group members; TCI, Team Climate Inventory.

### 3.2 Participatory process

In the interviews, professionals indicated they valued the opportunity to participate in the improvement process. They felt their feedback throughout the process was taken seriously, the improvement activities were tailored to their needs and they were satisfied about the impact of the activities on their way of working. Steering group members had more ambivalent views. While some perceived the implemented improvement activities to be meaningful, albeit on a small scale, others felt they could have gotten more out of the improvement process. Minutes of steering group meetings and research notes reflected that the steering group's initial ideas were more ambitious, both in terms of scope (i.e. health and social care working with one shared model for care) and scale (i.e. improvements implemented throughout the entire region). However, the research team's efforts to convert the steering group's ideas and ambitions into concrete and tangible improvement activities resulted in a more pragmatic and small‐scale approach. The additional refinement of the activities during their implementation implied another step further from the steering groups' initial ambition, causing some steering group members to indicate that the project had lost focus on these aspects and that they had not always felt involved in these decisions (see Table 3, quote 3).

### 3.3 Factors that enabled the improvement process

Several factors were identified that enabled the improvement process in West‐Friesland (see Table 5). For readability purposes, we sorted factors into different themes on the micro, meso and macro levels of the health and social care system. In reality, these factors were often interrelated. On the micro level, professionals indicated in interviews that the research team who organised and facilitated the intervision meetings had been important to the process. According to professionals, the research team motivated them and took action upon their feedback, ensuring that they felt respected and that the meetings had been valuable to them. Furthermore, professionals explained that the research team created a safe environment for them to discuss their experiences and issues. The informal setting of the meetings contributed to this and encouraged the development of personal relationships and trust, which was a recurring theme in the data. Both steering group members and professionals indicated that collaboration begins with trust and understanding (see Table 3, quote 4). Other factors that enabled implementation of the improvement activities included the motivation and commitment and the multidisciplinary background of professionals participating in intervision meetings and workplace visits. Professionals also experienced a sense of mutual gain from participating, as they felt their attendance was not only valuable to themselves, but to the other participants as well.

**Table 5:** Factors enabling and constraining the improvement process in West‐Friesland

	Enabling factors	Constraining factors
Micro (operational) level	Facilitation of intervision meetings Informal setting Safe environment Broad composition of professionals participating in meetings Commitment of participating professionals Personal relationships and trust among professionals from different organisations	Discrepancy between goal of intervision meetings and needs of participating professionals Lack of continuity in intervision meeting attendance Lack of time due to staff shortages and high case load
Meso (managerial) level	Process facilitation and management Broad composition of steering group Commitment on managerial level of participating organisations Personal relationships and trust among managers from different organisations Shared sense of urgency	Lack of continuity in steering group meeting attendance Lack of ownership and accountability among steering group members Conflicting organisational cultures and interests
Macro (regional and national) level	Regional policy to improve collaboration Complementary collaborative initiatives in the region	Limiting privacy regulations Lack of shared IT‐system Separate payment systems Lack of shared accountability

Note

The enabling and constraining factors presented in this table were identified based on interviews, meeting notes and field notes.

On meso level, the commitment and support of participating managers enabled professionals to invest time in the improvement activities. Furthermore, interviews with steering group members revealed that their meetings had helped them to develop shared vision regarding the core objectives of care and support for older people. Factors on the macro‐level, such as regional policies and collaborative initiatives across organisations, also facilitated this process (see Table 3, quote 5). Moreover, steering group members valued the presence of a representative of the regional advocacy organisation for older people. According to them, this representative's perspective helped to create a shared sense of urgency, and this binding factor was important in the development of trust and understanding on managerial level. These patterns in the qualitative data were also observed in the TCI scores (Table 4), which showed that improved total scores were largely due to improvements in participative safety on steering group member level. Additionally, steering group members indicated that the research team's role as process facilitators and managers had been important to the progress of the improvement process.

### 3.4 Factors that constrained the improvement process

Factors that constrained the improvement process were also distinguished into different themes on micro, meso and macro level (See Table 5). On the micro level, professionals indicated in interviews that the goals and timeline of the improvement activities had not been clear to them during the first two intervision meetings, which was related to the discrepancy between the initial meeting goals and professionals' own goals and needs. In addition, meeting and field notes indicate that continuity in attendance of the intervision meetings fluctuated especially during the first intervision meetings. The group interview revealed that this lack of continuity was perceived as disruptive by the regular attendees, as it hindered the development of personal relationships, trust and participative safety. Furthermore, professionals indicated that due to staff shortages and high caseloads, it was difficult to invest enough time in the intervention.

On the meso level, the composition of the steering group also changed throughout the process. Meeting and field notes showed that attendance during steering group meetings fluctuated due to time constraints and job changes. Some members felt that at times, this resulted in less constructive meetings due to repetition or lack of understanding of the topics discussed (see Table 3, quote 6). Additionally, the research team observed little ownership for the improvement process among the steering group. Steering group members also indicated in interviews that they never felt accountable for the process and at times had been unclear about the progress of the improvement process and their tasks and responsibilities in it. Furthermore, steering group members indicated that the different organisations involved often have different cultures and incompatible interests, which made it difficult to come to tangible agreements.

On the macro‐level, several other constraining factors emerged from the data. Recurring themes in the interviews and meeting notes included the fragmentation between the health and social care systems, for example regarding financing, privacy regulations, a lack of a shared IT‐system and a lack of shared accountability across the collaborative partners. According to steering group members, these factors impeded collaboration agreements and made it difficult to implement improvement activities on a larger scale. Professionals mainly mentioned fragmented financing and privacy regulations as issues that made their day‐to‐day work difficult, although at the same time they indicated that they often found ways to work around these issues.

## 4 DISCUSSION

This case study described the experiences with an improvement process of an existing integrated care initiative in the Netherlands. The implemented improvement activities enhanced communication, trust and understanding between health and social care professionals, and were considered valuable by the participants. In addition, our study revealed the challenges related to such a process. Different organisational cultures and interests and lack of ownership and accountability among managers hindered the improvement process, whereas issues such as staff shortages, time constraints and privacy regulations made it difficult to implement improvements on a larger scale. Still, the participatory approach encouraged the development of shared goals, vision and sense of urgency both on the managerial and operational levels. Overall, many factors were identified that influenced the improvement process. Some of these were related to the *content* of the improvement activities, whereas others were related to the actual *improvement process* or to the *methodological* *approach* to this process. Although these factors operated on different levels of the health and social care system, they were often interrelated.

The intended *content* of the improvement activities contained elements to improve both person‐centredness of care delivery as well as collaboration between different stakeholders in health and social care. Ultimately, however, the implemented improvement activities mainly targeted collaboration, as the initial aim of improving person‐centredness of care delivery appeared to be unfeasible. Previous studies suggest that communication, understanding and trust are important prerequisites for interprofessional collaboration (Borgermans et al., 2017; Mulvale, Embrett, & Razavi, 2016; Xyrichis & Lowton, 2008), and are part of a transformational process that needs to be established before one can change actual care delivery processes (Manojlovich et al., 2015). This could explain why activities targeting person‐centredness were not in line with professionals' needs at the time of the improvement process; these activities targeted the care delivery process, whereas the prerequisites for interprofessional collaboration still needed to be established.

To achieve and improve prerequisites such as communication, understanding and trust, active investment in teambuilding and professional development is necessary (Mager & Lange, 2014; McEwan, Ruissen, Eys, Zumbo, & Beauchamp, 2017)—after all, as we observed in our study, unknown makes unloved. This and other studies have shown that dedicating time to engage in shared reflection and mutual learning is a useful approach to establish this (Jones & Jones, 2011; Kassianos et al., 2015). Furthermore, having a facilitator to guide this group process has been shown to ensure a safe environment (Sorensen, Stenberg, & Garnweidner‐Holme, 2018). Policy makers and professionals aiming to improve collaboration in integrated care are therefore recommended to invest time and resources in activities targeting collaborative skills and interpersonal dynamics, in order to establish well‐functioning teams.

The experiences described in this case study confirm that improving an existing way of working is a complex and nonlinear *process*. Although existing models for change and improvement acknowledge the iterative and cyclical nature of these activities (van Dongen et al., 2018; Glasgow, 2013; Taylor et al., 2014), this study highlights that even the process within one iteration of goal setting, implementing, evaluating and refining is not straightforward. Several lessons for a successful improvement process can be learned from this case study. First, there should be time and space allocated to all steps of the improvement cycle, including the process of developing mutual trust and setting shared goals (Rycroft‐Malone et al., 2016). This case study showed that developing partnerships and shared goals is especially difficult and time‐consuming when the improvement process involves multiple organisations with different cultures and ambitions. Second, it is important to have an initiator or facilitator who functions as driving force and connector. In this case, the research team had this role, and other studies have shown similar positive experiences with such ‘champions’ who facilitate boundary spanning and help create team vision and a sense of urgency (Greenhalgh et al., 2004; Mulvale et al., 2016; Rycroft‐Malone et al., 2016). However, the potential of such a facilitator is also dependent on their credibility, authority and a supportive environment (Rycroft‐Malone et al., 2018). Finally, developing and implementing tangible improvements to integrated care requires commitment from stakeholders on multiple levels, since sustainable improvements need adjustments on the operational, managerial and administrative levels of the health and social care systems (Borgermans et al., 2017; Mulvale et al., 2016). Our study showed that, as the improvement activities narrowed and became more pragmatic, professionals became more committed to the improvement process while the steering group members' commitment and engagement decreased. This discrepancy suggests that multiple improvement cycles with more rapid evaluation and learning mechanisms may be needed to incentivise continued commitment on multiple levels (Rycroft‐Malone et al., 2016).

Some observations regarding the use of participatory *methods* to improve and evaluate integrated care practice can also be made based on this case study. In line with the EIT and other models (Glasgow et al., 2012; Horowitz, Robinson, & Seifer, 2009; Jull, Giles, & Graham, 2017), the needs and priorities of local stakeholders were the starting point of the improvement process. This approach facilitated the establishment of partnerships between researchers and stakeholders, but also between stakeholders themselves, and it stimulated stakeholders to challenge their established mind‐set and views (Eyre et al., 2017; Martin Fortin & Moira, 2016). Theoretically, another key aspect of participatory research is that researchers and local stakeholders share decision‐making, ownership and accountability for the process (Blevins, Farmer, Edlund, Sullivan, & Kirchner, 2010; Horowitz et al., 2009; Jull et al., 2017; Viswanathan et al., 2004). Unfortunately, this extent of participation was not achieved in this case. In fact, the research team functioned as initiators and driving force of the project and, unable to transfer ownership to local stakeholders, their role remained prominent until the end.

Several explanations could be provided for these experienced difficulties regarding ownership. First, stakeholders' level of participation was influenced by the reality of day‐to‐day practice. Contextual factors such as financing problems, new privacy regulations and staffing shortages meant that stakeholders were not completely willing or able to commit to the improvement project at that time. Second, the research team experienced tension between tailoring the improvement process to stakeholders' needs and priorities and complying with the requirements set by the larger European project it was part of (de Bruin et al., 2018). For instance, as the stakeholders' initial ambitions were not compatible with the scale and timeline of the European project, several pragmatic decisions were made for which stakeholders may not have felt ownership. Future research should consider this tension between the flexibility needed for participatory approaches and the rigour and structure associated with traditional research projects. Expectation management towards stakeholders regarding the timeline and scale of future projects may additionally help to improve commitment (Allen et al., 2017; Horowitz et al., 2009; Viswanathan et al., 2004).

### 4.1 Methodological considerations

Data were collected among participating stakeholders. As this number was limited, our study sample was small. Staff changes and drop out of professionals during the project further affected the study sample size. However, data triangulation revealed similar patterns across different sources and types of data. Therefore, although results from each individual data source should be treated with caution, the overall picture provided by this case study approach is robust. Furthermore, the process‐oriented and multimethod approach provided detailed insights into the complexity of improving integrated care (Greenhalgh et al., 2004). Still, this case study only provides insight into experiences and outcomes at the level of service providers. Given the short implementation period, it was not feasible or functional to assess effects at the level of older people receiving care and support. The question of whether and how improvements to integrated care affect the experiences of service recipients might be addressed in future research.

## 5 CONCLUSION

On the basis of this study, we conclude that the participatory method is a promising approach to improve integrated care practice. However, achieving true participatory research in the reality of day‐to‐day practice is difficult, as many factors can influence stakeholders' willingness and ability to commit to this participatory role. In addition, researchers need to find a balance between the flexibility needed for participatory research and the structured context in which such research projects are usually embedded. Nevertheless, the participatory approach allows for the development of partnerships and shared goals on multiple levels of organisations. Since improving integrated care starts with improving interprofessional collaboration, establishing relationships, trust and communication between stakeholders are important prerequisites that should not be overlooked. Rather, they are the necessary foundation based on which further improvements to integrated care may be developed.

## CONFLICT OF INTEREST

The authors declare that they have no competing interests.

## AUTHORS' CONTRIBUTIONS

As members of the SUSTAIN consortium, SdB, CB, GN, AS and ML were involved in the development of the SUSTAIN methodology. ML, MB and CB facilitated the case study presented in this paper. ML collected the data, ML, LL and AS analysed the data and all authors together interpreted the data. ML drafted the manuscript and MB, LL, AS, GN, CB and SdB critically revised the manuscript. All authors approved of the final manuscript.
