# Supplementary material for: Unknown makes unloved—A case study on improving integrated health and social care in the Netherlands using a participatory approach
Source: Health Soc Care Community. 2019 Nov 27;28(2):670–80. doi: 10.1111/hsc.12901 (PMC7028071; doi:10.1111/hsc.12901)
Supplement: Supplementary file 1 [file HSC-28-670-s001.pdf]

Appendix 1 – Raw data on team Climate Inventory

Table A1. Team Climate Inventory individual and overall mean scores

	**Baseline (mean scores, range 1-5)**					**Follow-up (mean scores, range 1-5)**				
	Total TCI	Vision	Participative safety	Task orientation	Support for innovation	Total TCI	Vision	Participative safety	Task orientation	Support for innovation
**Professionals**										
NLP001	3.79	3.75	3.75	4.0	3.67	4.21	4.25	4.25	4.0	4.33
NLP002	3.69	4.0	3.75	3.0	3.67	3.86	4.0	4.0	3.33	4.0
NLP003	4.86	4.75	5.0	5.0	4.67	n.i.	n.i.	n.i.	n.i.	n.i.
NLP004	3.0	3.75	2.25	2.67	3.33	n.i.	n.i.	n.i.	n.i.	n.i.
NLP005	1.79	1.50	2.50	1.0	2.0	3.14	3.75	2.75	3.0	3.0
NLP006	2.93	3.25	3.0	3.0	2.33	3.86	3.75	4.25	3.67	3.67
NLP007	n.c.	n.c.	n.c.	n.c.	n.c.	3.29	2.75	4.0	3.33	3.0
NLP008	n.i.	n.i.	n.i.	n.i.	n.i.	2.07	2.25	1.5	2.67	2.0
Total score	3.3	3.8	3.4	3.0	3.5	3.6	3.8	4.0	3.3	3.3
**Steering group**										
NLM001	3.93	4.5	4.0	3.33	3.67	3.93	4.5	4.25	3.33	3.33
NLM002	2.71	3.25	2.5	1.67	3.33	4.0	4.0	.	.	.
NLM003	2.79	3.0	2.75	2.33	3.0	3.5	3.75	3.75	3.0	3.33
NLM004	3.07	4.5	1.75	3.0	3.0	n.c.	n.c.	n.c.	n.c.	n.c.
NLM005	n.i.	n.i.	n.i.	n.i.	n.i.	3.79	3.75	4.25	3.33	3.67
Total score	2.9	3.9	3.6	2.7	3.2	3.9	3.9	4.3	3.3	3.3
**Overall total score**	3.0	3.8	2.9	3.0	3.3	3.8	3.8	4.0	3.3	3.3

Note: this table presents individual scores on team climate, collected among steering group members and professionals at the start and end of implementation of the improvement project.
Abbreviations: TCI, Team Climate Inventory; n.c., not completed - this participant did not complete the questionnaire at the time; n.i., not involved - this participant was not involved in the project at the time.
